# Low diagnostic yield of routine preoperative chest CT in patients undergoing partial nephrectomy: A national cohort study using UroCCR data (UroCCR n°216)

**DOI:** 10.1002/bco2.70203

**Published:** 2026-09-09

**Authors:** Ali Bourgi, Gaëlle Margue, Pierre Bigot, Norbert De Brek, Louis Surlemont, Martin Lorette, Nicolas Branger, Thomas Prudhomme, Aurélien Adypagavane, Véra Chatain, Thibaut Waeckel, Alexis Fontenil, Victor Gaillard, Jean‐Jacques Patard, Constance Michel, Julien Guillotreau, Olivier Belas, Maxime Vallée, Stéphane De Vergie, Jean‐Baptiste Beauval, Clément Sarrazin, Benjamin Delattre, Frédéric Panthier, Lionel Hoquetis, Romain Boissier, Julien Anract, Marie Chicaud, Alexandre Marsaud, Richard Mallet, Quentin‐Côme Le Cerlc, Khalil Chalhoub, Idir Ouzaid, Jean‐Christophe Bernhard, Franck Bruyère

**Affiliations:** ^1^ CHRU Tours Service d'Urologie Tours France; ^2^ Hôpital Pellegrin – CHU Bordeaux/Icare Bordeaux Service d'Urologie Bordeaux France; ^3^ CHU Angers Service d'Urologie Angers France; ^4^ Hôpital Européen Georges Pompidou – APHP Service d'Urologie Paris France; ^5^ CHU Rouen Service d'Urologie Rouen France; ^6^ CHU Lille Service d'Urologie Lille France; ^7^ Institut Paoli‐Calmettes Service d'Urologie Marseille France; ^8^ CHU Rangueil – Toulouse Service d'Urologie Toulouse France; ^9^ Hôpital Bicêtre – APHP Service d'Urologie Le Kremlin‐Bicêtre France; ^10^ Hôpital Lyon Sud – Hospices Civils de Lyon Service d'Urologie Oullins‐Pierre‐Bénite France; ^11^ CHU Caen Normandie Service d'Urologie Caen France; ^12^ CHU Nîmes Service d'Urologie Nîmes France; ^13^ CHRU Strasbourg Service d'Urologie Strasbourg France; ^14^ CH Mont‐de‐Marsan Service d'Urologie Mont‐de‐Marsan France; ^15^ Hôpital Paris Saint‐Joseph Service d'Urologie Paris France; ^16^ Clinique Pasteur – Toulouse Service d'Urologie Toulouse France; ^17^ Pôle Santé Sud – Le Mans Service d'Urologie Le Mans France; ^18^ CHU Poitiers Service d'Urologie Poitiers France; ^19^ CHU Nantes Service d'Urologie Nantes France; ^20^ Clinique La Croix du Sud Service d'Urologie Quint‐Fonsegrives France; ^21^ CHU Grenoble Service d'Urologie Grenoble France; ^22^ CHU Dijon Service d'Urologie Dijon France; ^23^ Hôpital Tenon – APHP Service d'Urologie Paris France; ^24^ Clinique Nantes Atlantis – Saint‐Herblain Service d'Urologie Saint‐Herblain France; ^25^ CHU La Conception – Marseille (APHM) Service d'Urologie Marseille France; ^26^ Hôpital Cochin – Port‐Royal – APHP Service d'Urologie Paris France; ^27^ CHU Limoges Service d'Urologie Limoges France; ^28^ Clinique Saint‐George Service d'Urologie Nice France; ^29^ Hôpital Privé Francheville Service d'Urologie Périgueux France; ^30^ Clinique Santé Atlantique – Nantes Service d'Urologie Saint‐Herblain France; ^31^ CH Kourou Service d'Urologie Kourou France; ^32^ Hôpital Bichat – APHP Service d'Urologie Paris France

**Keywords:** chest CT, diagnostic imaging, partial nephrectomy, pulmonary metastasis, renal cell carcinoma, risk‐adapted strategy

## Abstract

**Objective:**

The aim of this study was to evaluate the diagnostic yield and downstream clinical impact of routine preoperative chest CT in patients undergoing partial nephrectomy (PN) for localized renal cell carcinoma.

**Methods:**

We conducted a retrospective multicentre study using data from the French Urological Cancer Comprehensive Cohort (UroCCR) registry. Adult patients who underwent PN for localized renal masses between 2010 and 2024 and had preoperative chest CT within 30 days before surgery were included. The primary endpoint was the detection rate of synchronous pulmonary metastases. Secondary endpoints included characteristics of metachronous metastases and diagnostic or scheduling consequences of preoperative imaging.

**Results:**

Among 7351 patients, 6679 underwent surgery and 5016 had a preoperative chest CT. Of these, 4483 (89.4%) had clinical T1 tumours. Synchronous pulmonary metastases were identified in only 17 patients (0.38%), all with larger or higher grade tumours. These patients had significantly higher rates of positive surgical margins (17.6% vs. 6.1%, *p* = 0.04), but no significant difference in age, sex or comorbidity profile. Among 6075 patients with postoperative follow‐up, 191 (3.14%) developed metachronous pulmonary metastases. Of these, 14 (7.3%) had a previously negative preoperative CT, with a median time to progression of 52.6 months. Importantly, 166 (3.7%) patients underwent additional, ultimately non‐contributive thoracic investigations, and 2.1% experienced surgical delays due to incidental or equivocal CT findings.

**Conclusions:**

The diagnostic yield of routine preoperative chest CT in patients with clinical T1 RCC is exceedingly low (<0.5%). These data support the omission of routine chest CT in asymptomatic, low‐risk cT1 RCC, potentially sparing over 95% of patients from unnecessary imaging, additional tests and surgical delays. A risk‐adapted, symptom‐guided strategy may optimize patient care while reducing radiation exposure and healthcare costs.

## 1 INTRODUCTION

Renal cell carcinoma (RCC) represents approximately 2%–3% of all adult malignancies, with a rising incidence primarily attributed to the increased use of cross‐sectional imaging that often detects small, asymptomatic renal masses incidentally.^1^, ^2^ As a result, many tumours are diagnosed at an early stage, particularly clinical T1 tumours, for which partial nephrectomy (PN) has become the preferred surgical approach. PN offers comparable oncologic outcomes to radical nephrectomy while preserving renal function, reducing cardiovascular morbidity and maintaining quality of life.^3^ In parallel, recent studies have highlighted variability in adherence to imaging recommendations in patients with cT1 renal masses.^4^

Preoperative imaging plays a crucial role in staging RCC and in evaluating for distant metastases, most frequently located in the lungs, followed by bone and liver.^5^ Although chest radiography has traditionally been used for asymptomatic patients, chest computed tomography (CT) is increasingly employed—even in patients with low‐risk tumours—due to its higher sensitivity. However, the clinical utility of routine chest CT in patients undergoing PN for localized RCC has been increasingly questioned.

Multiple recent studies have demonstrated that the diagnostic yield of chest CT in detecting synchronous pulmonary metastases in this population is exceedingly low, often below 1%.^6^, ^7^ Furthermore, chest CT may identify incidental pulmonary nodules of uncertain significance, leading to additional testing, patient anxiety, delayed surgery and higher healthcare costs without clear improvement in patient outcomes.^8^, ^9^, ^10^

In response to these findings, international guidelines now advocate for a risk‐adapted approach to chest imaging. The European Association of Urology (EAU)^11^ and the American Urological Association (AUA)^12^ recommend limiting chest CT to patients with advanced‐stage tumours, respiratory symptoms, abnormal chest X‐ray or clinical suspicion of metastases.

Despite these recommendations, real‐world adherence remains variable. We hypothesized that the diagnostic yield of chest CT in clinical T1 RCC is negligible and that its routine use may delay curative surgery without improving outcomes.

This study aims to evaluate the diagnostic value of preoperative chest CT in a large national cohort of patients undergoing partial nephrectomy for localized RCC, to inform evidence‐based decision‐making and promote judicious use of diagnostic imaging.

## 2 METHODS

This study is a retrospective multicentre cohort analysis based on prospectively collected data from the Urological Cancer Comprehensive Cohort (UroCCR), a national French registry dedicated to urologic cancers. UroCCR is supported by the French National Cancer Institute (INCa) and includes standardized data from 38 academic and community centres.

The UroCCR registry received ethical approval from the Comité de Protection des Personnes Sud‐Ouest et Outre‐Mer III (decision number DC 2012/108) and a favourable opinion from the CCTIRS. It is registered as the UroCCR project (NCT03293563), which is IRB‐approved and holds CNIL authorization no. DR‐2013‐206.

In addition, the registry operates under the CNIL decision no. DT‐2024‐027 of 31 December 2024, authorizing the Bordeaux University Hospital to implement automated data processing for the creation of the UroCCR health data warehouse (authorization request no. 2231991).

The UroCCR registry incorporates standardized data collection procedures, periodic data audits, automated consistency checks and multicentre quality control measures to ensure accuracy and completeness of oncologic outcomes.

All patients received oral and written information regarding the objectives and methodology of the UroCCR project, and written informed consent was obtained at the time of inclusion.

We included adult patients (≥18 years) who underwent partial nephrectomy between January 2010 and December 2024 for a radiologically suspicious renal mass. Patients with metastatic disease diagnosed prior to chest imaging, recurrent or bilateral tumours, missing chest imaging data or who underwent radical nephrectomy or ablation were excluded.

This study was specifically designed to evaluate the diagnostic yield of preoperative chest CT in patients selected for nephron‐sparing surgery. Therefore, the cohort represents a surgically selected population with presumed lower metastatic risk compared to the overall population of patients with cT1 renal masses.

Collected data included demographics (age, sex and BMI), comorbidities (Charlson Comorbidity Index), tumour features (clinical and pathological stage, size, histological subtype and laterality), surgical parameters (approach, ischemia time and blood loss) and preoperative imaging records.

Synchronous pulmonary metastasis was defined as radiologically suspected metastatic lung lesions identified on preoperative chest CT performed within 30 days prior to surgery. Synchronous pulmonary metastases were defined based on radiologic features consistent with metastatic disease and confirmed through multidisciplinary tumour board consensus. Histological confirmation was obtained when clinically indicated.

The primary outcome was the diagnostic yield of routine preoperative chest CT, defined as the number of patients with confirmed synchronous pulmonary metastases divided by the total number of patients who underwent chest CT. Secondary endpoints included comparisons of demographic, clinical and pathological characteristics between patients with and without metastatic findings on imaging.

Statistical analysis was conducted using R software (version 4.2.1). Continuous variables were presented as median values with interquartile ranges (IQR) and compared using the Mann–Whitney *U* test. Categorical variables were expressed as frequencies and percentages, with comparisons made using the chi‐squared test or Fisher's exact test when expected cell counts were low. Given the small number of patients with confirmed pulmonary metastasis (*n* = 17), only descriptive and univariate analyses were performed to avoid overfitting and unstable estimates in multivariable models.

A two‐sided *p*‐value < 0.05 was considered statistically significant.

## 3 RESULTS

**FIGURE 1:**
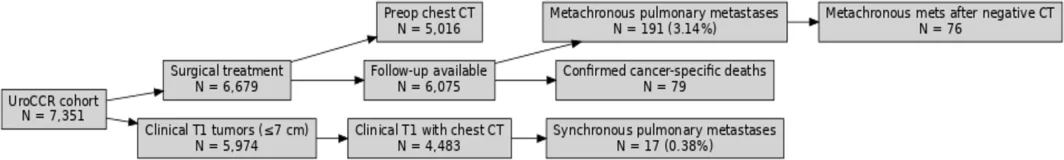
Flowchart of patient selection from the Urological Cancer Comprehensive Cohort (UroCCR).

A total of 7351 patients were included in the national UroCCR cohort. Among them, 6679 patients underwent surgical treatment for a renal mass, and 5016 had a documented preoperative chest computed tomography (CT). Tumour size data were available for 7345 patients, among whom 5974 (81.3%) had clinical T1 tumours (≤7 cm). Within this subgroup, 4483 patients underwent preoperative chest CT imaging (Figure 1). Median follow‐up among patients with available surveillance was 48 months (IQR 24–86).

Pathologic upgrading from clinical T1 to pathological ≥T3 occurred in 238 patients (5.3%). Upgrading was observed in three of 17 patients (17.6%) with synchronous metastases compared to 235 of 4466 (5.3%) in the non‐metastatic group (*p* = 0.06).

### 3.1 Synchronous pulmonary metastases

Among the 4483 patients with clinical T1 tumours and preoperative chest CT, 17 (0.38%) had radiologically confirmed synchronous pulmonary metastases. All 17 patients subsequently underwent surgical resection of their primary renal tumour, and their lung lesions were deemed resectable and managed with curative intent (surgery or stereotactic radiotherapy).

Baseline characteristics of patients with versus without synchronous pulmonary metastases are detailed in Table 1. There were no statistically significant differences in age (mean 64.3 vs. 61.8 years, *p* = 0.48), sex distribution (70.6% vs. 65.3% male, *p* = 0.59), BMI (27.1 vs. 27.4 kg/m^2^, *p* = 0.72) or comorbidities such as diabetes and pulmonary disease. All 17 patients with synchronous metastases had clear cell renal cell carcinoma (ccRCC), compared to 92.3% in the non‐metastatic group (*p* = 0.06). Tumour size was slightly larger in the metastatic group (median 3.2 vs. 2.82 cm), although this was not statistically significant (*p* = 0.25). Positive surgical margins were more common in the metastatic group (17.6% vs. 6.1%, *p* = 0.04).

**TABLE 1:** Comparison of clinical and pathological characteristics between patients with and without synchronous pulmonary metastases on preoperative chest CT.

Variable	With metastases (*n* = 17)	Without metastases (*n* = 4466)	*p*
Number of patients	17	4466	‐
Sex (male), *n* (%)	12 (70.6%)	2917 (65.3%)	0.59
Age, years (mean ± SD)	64.3 ± 8.7	61.8 ± 11.2	0.48
BMI, kg/m^2^ (mean ± SD)	27.1 ± 4.2	27.4 ± 5.1	0.72
			0.157
ASA 1, *n* (%)	2 (11.8%)	1130 (25.3%)	
ASA 2, *n* (%)	8 (47.1%)	2370 (53.1%)	
ASA 3, *n* (%)	6 (35.3%)	894 (20.0%)	
ASA 4, *n* (%)	1 (5.9%)	72 (1.6%)	
Smoking, *n* (%)	6 (35.3%)	1115 (25.0%)	0.12
Diabetes, *n* (%)	3 (17.6%)	785 (17.6%)	0.95
Pulmonary disease, *n* (%)	2 (11.8%)	540 (12.1%)	0.68
Median tumour size (cm)	3.2	2.82	0.25
Tumour side (left), *n* (%)	11 (64.7%)	2208 (49.4%)	0.19
Histology (clear cell), *n* (%)	17 (100%)	4122 (92.3%)	0.26
Median RENAL Score	7	7	0.87
ISUP grade			0.107
ISUP Grade 1, *n* (%)	0 (0.0%)	432 (9.7%)	
ISUP Grade 2, *n* (%)	3 (17.6%)	1570 (35.2%)	
ISUP Grade 3, *n* (%)	9 (52.9%)	1472 (33.0%)	
ISUP Grade 4, *n* (%)	5 (29.4%)	802 (18.0%)	
Positive surgical margins, *n* (%)	3 (17.6%)	273 (6.1%)	0.04
Pathological stage ≥ pT3	3 (17.6%)	235 (5.3%)	0.06

Missing data were limited across variables: BMI (1.9%), smoking status (2.5%), RENAL score (6.1%), tumour size (0.8%) and ISUP grade (1.2%). No missing data were observed for age, sex or ASA classification.

### 3.2 Metachronous pulmonary metastases

Among 6075 patients with available postoperative follow‐up data, 191 (3.14%) developed metachronous pulmonary metastases. Of these, 76 had undergone preoperative chest CT that was initially negative, suggesting true postoperative disease progression rather than missed synchronous metastasis. In total, 79 patients in the cohort died from renal cancer, as documented in follow‐up.

### 3.3 Delays and additional findings related to chest imaging

While the diagnostic yield of preoperative chest CT was low, incidental thoracic findings were frequent.

Among the 4483 cT1 patients who underwent preoperative chest CT, 166 patients (3.7%) required additional thoracic imaging or invasive work‐up (e.g., PET/CT, bronchoscopy or biopsy) due to indeterminate or benign‐appearing pulmonary nodules, ultimately found to be non‐metastatic.

Common benign lesions included granulomas, fibrotic scars and small inflammatory nodules.

Ninety‐four patients (2.1%) experienced delays in surgical scheduling due to these investigations and multidisciplinary tumour board re‐evaluations.

While the exact duration of delay could not be quantified, qualitative review indicated that such unnecessary diagnostic workups occurred in at least 5% of cases overall.

These additional investigations rarely altered surgical management, highlighting the low clinical yield and potential harms of routine preoperative chest CT in asymptomatic cT1 RCC.

**FIGURE 2:**
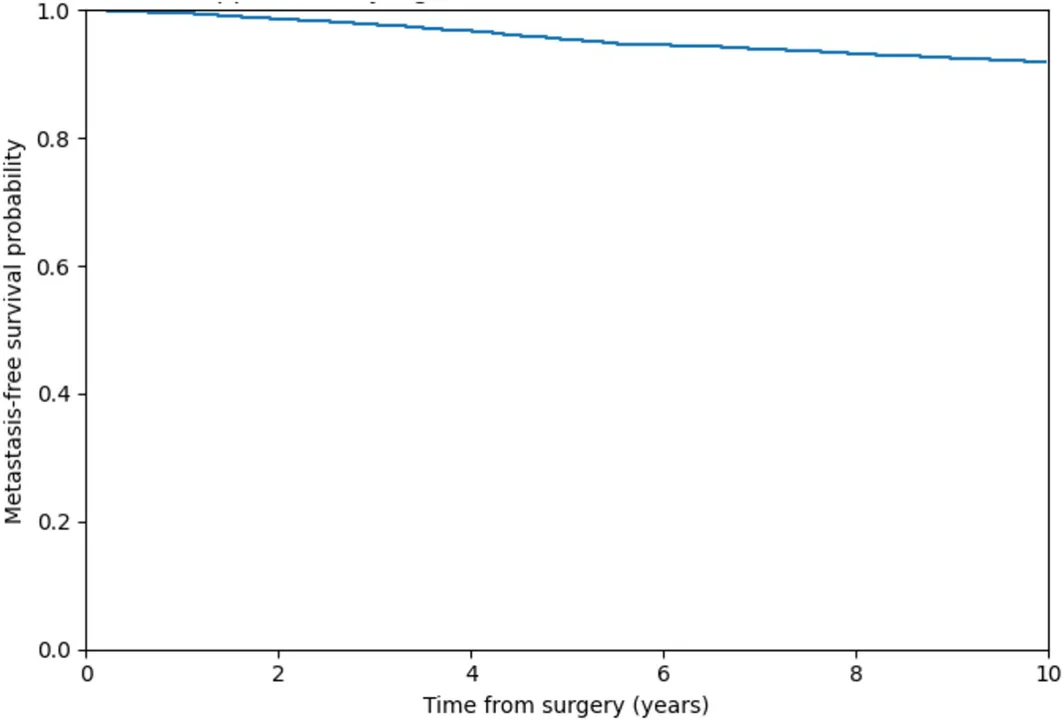
Kaplan–Meier curve showing metastasis‐free survival (including pulmonary and extrapulmonary metastatic events) among patients undergoing partial nephrectomy for clinical T1 renal cell carcinoma.

Overall metastasis‐free survival, including both pulmonary and extrapulmonary metastatic events, is shown in Figure 2. The estimated metastasis‐free survival rates were 99.6% at 1 year, 97.8% at 3 years and 95.4% at 5 years. Median metastasis‐free survival was not reached during follow‐up.

## 4 DISCUSSION

In this large national cohort of 7351 patients with localized renal masses, including 6679 surgically treated cases and 4483 clinical T1 tumours with preoperative chest CT imaging, the diagnostic yield of routine thoracic imaging was exceptionally low. Only 17 cases (0.38%) of synchronous pulmonary metastases were identified among clinical T1 patients, and all were treated with curative intent. These findings reinforce prior evidence suggesting that routine preoperative chest CT has limited value in asymptomatic patients with small renal tumours.^6^, ^7^

Unlike previous single‐centre reports or insurance‐claims analyses, our study draws on data from the UroCCR national registry, a prospectively maintained, quality‐controlled cohort integrating accurate radiologic, surgical and histopathologic information from 38 French centres. This national design provides a uniquely reliable estimate of the real‐world diagnostic yield of preoperative chest CT in small renal tumours.

Routine chest CT is not without consequence. In our study, several patients experienced delays in definitive surgical treatment due to the need for additional investigations prompted by incidental findings. Furthermore, the low rate of metastatic disease detection raises questions regarding the cost‐effectiveness, radiation exposure and psychological burden associated with routine imaging, as well as its limited diagnostic performance in detecting clinically relevant disease.^9^, ^10^, ^13^

Our findings confirm that most synchronous metastases occur in patients with larger or higher grade tumours, aligning with risk factors previously identified in institutional series. The absence of metastatic disease in more than 99% of cT1 tumours indicates that routine chest CT provides negligible additional staging information in this population.

Importantly, histologic subtype may also play a role. All patients with synchronous metastases had ccRCC, a histology more prone to hematogenous spread. However, clear cell histology was also prevalent in non‐metastatic patients (92.3%), limiting its discriminative power.

Regarding metachronous metastases, 191 patients (3.1%) developed delayed pulmonary recurrence after surgery, including 76 with a previously negative preoperative chest CT. The median time to detection exceeded four years (52.6 months), suggesting that these were true postoperative progressions rather than initially undetected disease. This supports a shift toward postoperative surveillance tailored to risk, rather than routine preoperative staging in all cT1 tumours.^14^

Taken together, these findings underscore the limited utility of routine preoperative chest CT in the majority of patients with clinical T1 RCC. Selective imaging strategies based on tumour size, grade and clinical risk factors are supported by both our results and prior models such as the Larcher nomogram.^15^ Furthermore, the low incidence of metachronous metastasis in patients with small, well‐differentiated tumours and negative margins suggests that postoperative surveillance protocols should also be personalized.

These data strengthen the case for a risk‐adapted imaging approach, consistent with EAU and AUA recommendations.^11^, ^12^ Based on our findings, preoperative chest CT should be reserved for patients with:

Tumour size >7 cm (≥cT2), aggressive or non‐clear cell histologic variants or clinical or radiographic suspicion of pulmonary disease (symptoms or abnormal chest X‐ray).

Such a selective strategy could safely spare >95% of cT1 RCC patients from unnecessary imaging and its downstream consequences, while maintaining appropriate staging in high‐risk disease.

Our study has limitations inherent to its retrospective design, including potential selection bias and missing data on performance status and laboratory results. Moreover, uniformity of postoperative imaging could not be fully verified across centres. Nevertheless, UroCCR incorporates periodic data audits, double data entry and automated consistency checks, ensuring a high level of data accuracy and completeness.

Importantly, our cohort was restricted to patients undergoing partial nephrectomy, which inherently represents a selected subset of renal tumours with favourable anatomy and oncologic characteristics. Patients with larger, anatomically complex or clinically aggressive tumours are more likely to undergo radical nephrectomy or systemic therapy, potentially resulting in an underestimation of the true prevalence of synchronous metastases in the broader population of cT1 renal masses.

In conclusion, both synchronous and metachronous pulmonary metastases are rare among patients with clinical T1 renal tumours. These results, derived from a large, prospectively maintained national cohort, strongly support the omission of routine preoperative chest CT in asymptomatic, low‐risk patients.

A risk‐based algorithm, incorporating tumour size, histologic subtype and clinical presentation, may guide smarter imaging practices and inform future updates of international guidelines.

## 5 CONCLUSION

In this large national cohort of patients undergoing partial nephrectomy for localized RCC, the diagnostic yield of routine preoperative chest CT was exceedingly low, with synchronous pulmonary metastases detected in only 0.38% of cases. These results confirm that chest CT offers limited clinical benefit in asymptomatic patients with low‐risk tumours (≤4 cm, cT1 stage and no systemic symptoms). Our findings reinforce the need for a risk‐adapted imaging approach, as recommended by international guidelines, to avoid unnecessary testing, minimize treatment delays and ensure more efficient use of healthcare resources.

## AUTHOR CONTRIBUTIONS

All authors contributed to the study conception and design. Material preparation, data collection and analysis were performed by Ali Bourgi and Franck Bruyere. The first draft of the manuscript was written by Ali Bourgi, and all authors commented on previous versions of the manuscript. All authors read and approved the final manuscript.

## CONFLICT OF INTEREST STATEMENT

The authors declare no conflicts of interest.
